# Fetal Growth Restriction Is Associated with Pregnancy Associated Plasma Protein A and Uterine Artery Doppler in First Trimester

**DOI:** 10.3390/jcm12072502

**Published:** 2023-03-26

**Authors:** Stephanie Springer, Katharina Worda, Marie Franz, Eva Karner, Elisabeth Krampl-Bettelheim, Christof Worda

**Affiliations:** 1 Department of Obstetrics and Gynecology, Medical University of Vienna, 1090 Vienna, Austria; 2 Department of Gynecology and Obstetrics, University Hospital, LMU Munich, 81377 Munich, Germany

**Keywords:** fetal growth restriction, uterine artery Doppler, PAPP-A, first-trimester screening

## Abstract

Fetal growth restriction (FGR) is a major cause of stillbirth and poor neurodevelopmental outcomes. The early prediction may be important to establish treatment options and improve neonatal outcomes. The aim of this study was to assess the association of parameters used in first-trimester screening, uterine artery Doppler pulsatility index and the development of FGR. In this retrospective cohort study, 1930 singleton pregnancies prenatally diagnosed with an estimated fetal weight under the third percentile were included. All women underwent first-trimester screening assessing maternal serum pregnancy-associated plasma protein A (PAPP-A), free beta-human chorionic gonadotrophin levels, fetal nuchal translucency and uterine artery Doppler pulsatility index (PI). We constructed a Receiver Operating Characteristics curve to calculate the sensitivity and specificity of early diagnosis of FGR. In pregnancies with FGR, PAPP-A was significantly lower, and uterine artery Doppler pulsatility index was significantly higher compared with the normal birth weight group (0.79 ± 0.38 vs. 1.15 ± 0.59, *p* < 0.001 and 1.82 ± 0.7 vs. 1.55 ± 0.47, *p* = 0.01). Multivariate logistic regression analyses demonstrated that PAPP-A levels and uterine artery Doppler pulsatility index were significantly associated with FGR (*p* = 0.009 and *p* = 0.01, respectively). To conclude, these two parameters can predict FGR < 3rd percentile.

## 1. Introduction

Fetal growth restriction (FGR) is a condition in which the fetus does not achieve its predetermined growth potential and is described with an incidence of 10% of all pregnancies [1,2]. FGR is a major cause of stillbirth, perinatal morbidity and mortality, as well as poor neurodevelopmental outcomes in the offspring [3,4,5,6]. This is especially true for fetuses under the 3rd percentile have higher rates of intrauterine fetal demise [7]. Neurologic impairment can be found in 20–40% of children following a fetal growth restriction under the 5th percentile [8]. In addition, these children show higher risks of suffering from metabolic disorders and cardiovascular diseases due to fetal programming [9,10]. These risks are lower when FGR is detected prenatally [11]. Moreover, the risk for stillbirth is eight times higher in fetuses where FGR was not detected [4,12,13].

On the one hand, early prediction of FGR would be important to identify those fetuses that are at the highest risk and lead to appropriate monitoring and delivery timing. On the other hand, early administration of low-dose aspirin might prevent the occurrence of FGR [6,14].

Identifying pregnancies that are at risk for fetal growth restriction (FGR) at an early stage of gestation is a major challenge for fetal medicine. 

The etiologies of FGR include congenital fetal malformations, genetic disorders or infectious diseases. However, the major causes of FGR are placental abnormalities. Insufficient placental circulation can lead to inadequate nutrition of the fetus. The basis for initial villous angiogenesis in early placental development is the release of various growth factors. Therefore, hormones secreted by the placenta, such as progesterone and human chorionic gonadotropin (HCG), usually lead to a dilatation of the uteroplacental arteries. Placental pathologies leading to FGR can occur at any step of the complex placental development. Often, the placenta shows reduced volume, surface and vascularization in fetuses with growth restriction, which can be due to the malregulation of certain molecular pathways [15,16,17,18,19].

One approach to determine pregnancies at risk for FGR is to combine indicators of placental dysfunction, such as uterine artery Doppler ultrasound and maternal serum biomarkers [20,21,22,23]. At first trimester screening for aneuploidies, maternal age is combined with nuchal translucency and other ultrasound markers. Tested parameters also include maternal serum levels of pregnancy-associated plasma protein A (PAPP-A) and free beta-human chorionic gonadotropin (beta-hCG) for risk calculation [24,25,26].

PAPP-A is a metzincin superfamily metalloprotease in the insulin-like growth factor (IGF) system. PAPP-A increases IGF bioavailability and mitogenic effectiveness in vitro through regulated cleavage of IGF-binding [27,28]. It is well known that low maternal serum PAPP-A levels in the first trimester are associated with poor fetal growth and development of pregnancy-induced hypertension, preeclampsia and stillbirth [29,30,31,32,33,34,35,36,37,38,39,40]. Very low PAPP-A levels are frequently related to adverse pregnancy outcomes [41]. The exact mechanism is not well understood, but studies suggest that low PAPP-A levels may indicate an inadequate trophoblast invasion [26]. The low positive predictive value of PAPP-A for subsequent adverse pregnancy outcomes limits the use of PAPP-A as a screening tool. 

Free beta-hCG is one of the hormones produced by the syncytiotrophoblast that leads to the activation of growth factors and cell proliferation [16]. The association between beta-hCG levels and adverse perinatal outcomes, including FGR, has been described several times [42,43]. Because of activating various biochemical pathways, different maternal serum levels may reflect different pathophysiological mechanisms relating to placental dysfunction and adverse perinatal outcome [44]. Two large screening studies showed that free-hCG levels were significantly decreased in pregnancies with FGR [45,46].

Uterine artery Doppler ultrasound at 20 weeks of gestation is used as a screening test for the prediction of FGR and preeclampsia. Pathological waveforms and the presence of a notch in the early diastole have been associated with the development of preeclampsia and poor fetal outcome [31]. Failure of trophoblast invasion during the first half of pregnancy may lead to reduced dilatation of the placental vessels, producing higher persistence. This can be detected by the waveform of the uterine artery, showing an increased pulsatility index (PI) [47,48]. Several studies have shown that already at the first-trimester screening, abnormal uterine artery flow is associated with FGR [46,49,50,51]. Uterine artery PI and first-trimester serum markers independently show an influence on FGR [43,52].

The nuchal translucency (NT) has been established as an important parameter in the risk assessment for aneuploidy [53]. The role of NT measurements in the risk stratification of FGR has been investigated by Poon et al. [45].

For the prediction of FGR, there have been some parameters described as helpful tools for the use of risk assessment. Most of these predictors arise from first-trimester screening for preeclampsia, which has been widely studied and is well-established [54]. A study by He et al. showed that the combination of uterine artery PI and maternal serum PAPP-A levels could predict FGR [25]. Other authors investigated similar values for fetuses small for gestation age (SGA) or late-onset FGR [55].

Poon et al. and Karagiannis et al. showed that a combination of maternal factors and biophysical and biochemical markers improve the prediction of FGR tremendously [45,46]. Our aim was to evaluate the effect of serum parameters together with uterine artery PI to assess the risk for FGR under the 3rd percentile at our center for fetal medicine.

## 2. Materials and Methods

This was a retrospective cohort study including 1930 consecutive women who underwent first-trimester combined screening for Down’s syndrome by measuring fetal nuchal translucency, maternal serum free beta-hCG and PAPP-A, as well as examinations of uterine artery Doppler. Additionally, second-trimester ultrasound screening for fetal abnormalities was performed. The study was conducted at the Department of Obstetrics and Gynecology, Division of Obstetrics and Feto-maternal Medicine, a tertiary referral center for fetal medicine over a period of two years (2019–2020). The annual birth rate in our department is about 3000. Only women with singleton pregnancies without chromosomal or structural anomalies and a liveborn baby after 23 weeks of gestation were included. Furthermore, pregnancies with missing birth data were not included. The study group consisted of pregnancies with FGR < 3rd percentile, whereas the control group consisted of neonates with normal birth weights. The women underwent assessment of the uterine artery Doppler in the first trimester, combined with the screening for chromosomal abnormalities. Gestational age was determined by menstrual history and confirmed or corrected at a first-trimester ultrasound scan. All measurements were performed according to the criteria of the Fetal Medicine Foundation between 11 + 0 to 13 + 6 weeks of gestation [56,57].

FGR was defined as birth weight less than the 3rd percentile corrected for fetal sex and parity, according to the International Federation of Gynecology and Obstetrics [15,25,58]. The ultrasound-estimated fetal weight (EFW) was calculated using Hadlock H1 method [59].

Ultrasound was performed with a transabdominal RM 6C convex probe of General Electric Healthcare E10 or Voluson E8 Expert equipped with a transabdominal RAB 4-8-D convex probe. The PI of the uterine artery was measured bilaterally by transabdominal ultrasound. At the first-trimester scan, a sagittal section of the uterus was obtained and internal cervical os was identified. Color flow mapping was used to identify each uterine artery along the side of the cervix at the level of the internal os. Pulsed wave Doppler was used with the sampling gate set at 2 mm, and the angle of insonation was less than 30°. Three consecutive waveforms were obtained, and the mean PI of the left and right arteries were calculated. Nuchal translucency (NT) was measured in fetal midsaggital plane with a fetal crown–rump length of 45 to 84 mm as required standard examination according to the Fetal Medicine Foundation certification [60].

Maternal serum parameters (PAPP-A, free beta-hCG) were biochemical analyzed by using a Kryptor Immunoassay Analyzer (Brahms, Berlin, Germany) and were expressed as multiple of the median (MoM) for gestational age.

Overall, we included 2563 women who had a singleton liveborn baby after 23 weeks without chromosomal or structural anomaly in the study. A total of 314 women (12.3%) did not undergo first-trimester screening for chromosomal abnormalities. Of the remaining 2249 women, 319 women (14.2%) were excluded due to missing variables. Therefore, 1930 women could be evaluated in the study. We obtained clinical data, including maternal, fetal, pregnancy and perinatal outcomes of all 1930 patients from electronic hospital records.

Data were analyzed by using SPSS 25.0 for Mac (SPSS Inc., Chicago, IL, USA). We performed a Kolmogorov–Smirnov test to verify the use of tests for normally distributed variables. Normally distributed variables are summarized as means (±standard deviation (SD) and categorical data as percentages. Chi-square test, *t*-test, and logistic multiple regression analyses were used accordingly. For the logistic regression analyses, the backward selection with likelihood ratio test was used. The better quality of fit of models was assessed using the Hosmer and Lemeshow test. We constructed a Receiver Operating Characteristics curve to calculate the sensitivity and specificity of early diagnosis of FGR. The cut-offs for mean PI and PAPP-A MoM were separately defined by using the Youden Index. Sensitivity, specificity and relative risk with a 95% confidence interval (95% CI) were evaluated. All tests were two-tailed, and *p*-value ≤ 0.05 was considered to be statistically significant.

The study was approved by the local ethics committee (IRB number 1083/14) and was performed according to the standards of the Helsinki Declaration.

## 3. Results

Overall, 1930 pregnant women were included in the study. The patient’s characteristics are shown in Table 1.

We have compared patient’s body mass index (BMI), maternal age, parity, PAPP-A, free beta-hCG, PI of the uterine artery and nuchal translucency at the first trimester in women at 11 + 0 − 13 + 6 weeks of pregnancy who delivered FGR babies and normal weight newborns.

Patients with FGR babies at birth had significantly lower PAPP-A levels (*p* < 0.001 by *t*-test) and higher uterine artery Doppler PI (*p* = 0.01 by *t*-test) at the first-trimester scan than women with normal-weight babies (Table 2). We could not achieve a significant result if we used PAPP-A < 0.4MoM, but we could demonstrate that PI of the uterine artery > 95% percentile was significantly associated with FGR (*p* = 0.01).

Nuchal lucency was slightly smaller in the FGR group but just not significant, with a p-value of 0.06. At the same time, the ß-HCG was only decently higher than that of the control group and, therefore, not significantly related to FGR (*p* = 0.42).

We identified PAPP-A and uterine artery Doppler PI in the first trimester to be significantly associated with FGR in univariate analyses. We performed logistic regression analyses with these two parameters. First-trimester serum PAPP-A levels and uterine artery Doppler PI were independently related to FGR (Table 3).

Newborns who were born before 32 weeks and were classified as FGR (n = 18) had significantly lower PAPP-A levels (0.65 ± 0.28 vs. 1.14 ± 0.58 MoM, *p* < 0.01) and had higher uterine artery Doppler PI at first trimester (2.14 ± 0.94 vs. 1.56 ± 0.48, *p* < 0.01) than newborns who were born after 32 weeks of gestation or were not classified as FGR.

We have tested the predictive power of PAPP-A and uterine artery Doppler PI in the first trimester for the development of FGR using the receiver-operating characteristics (ROC) curve. ROC curve showed the best test characteristics by calculating the highest Youden index when using the cut-off value of 1.15 MoM for PAPP-A and 1.7 for uterine artery Doppler PI in the first trimester. With these cut-off values, we received a sensitivity of 21% with a specificity of 62% (area under the curve (AUC) 0.3), which results in a negative predictive value of 93% for PAPP-A, and a sensitivity of 52% with a specificity of 66% (AUC 0.6), which results in a negative predictive value of 96% for uterine artery Doppler PI, respectively.

Furthermore, we have calculated a multinominal logistic regression analysis to demonstrate the effects of a combination of PAPP-A levels below 1.15 MoM and uterine artery Doppler PI above 1.7. Patients with both parameters positive had a significantly higher risk of developing FGR (*p* = 0.03, RR 1.93 (CI 1.16–3.2) than women with only one or no parameter positive.

## 4. Discussion

We evaluated maternal serum markers and uterine artery PI in the first trimester and found that PAPP-A and uterine artery Doppler PI were independently associated with FGR below the 3rd percentile.

It is well established that inadequate trophoblast invasion is associated with high-impedance uteroplacental flow at 11–14 weeks of gestation and with fetal growth restriction and preeclampsia. PAPP-A values below the 5th percentile in the first trimester have also been associated with higher rates of spontaneous fetal loss, low birth weight and preterm birth in several studies [29,30,31,41].

The FASTER trial evaluated in a study of 34,271 pregnancies the association of a fetal nuchal translucency ≥ 99th percentile, low PAPP-A and ß hCG levels and adverse pregnancy outcome. They found that PAPP-A below the 5% percentile was associated with spontaneous fetal loss ≤ 24 weeks of gestation, low birth weight, hypertensive disorders in pregnancy, preterm birth, stillbirth and placental abruption. As the sensitivity and positive predictive values were relatively low, the authors concluded that this parameter alone is a poor predictor for adverse pregnancy outcomes [61]. Pilalis et al. have examined the combination of uterine artery Doppler and maternal serum PAPP-A assessed in the first trimester. The combination of maternal history, abnormal uterine artery Doppler and low PAPP-A level at 11–14 weeks were better predictors for small gestational age fetuses than both markers alone [42].

A meta-analysis including 32 studies found a moderate association between low first-trimester PAPP-A and birth weight < 10th percentile and a strong association between low first-trimester PAPP-A and FGR < 1st percentile, but predictive values were poor [43]. This is in accordance with our results. Gaccioli et al. concluded from their results that although the low predictive value of PAPP-A limits its use as a screening tool for FGR, low PAPP-A levels could be considered to indicate closer monitoring of fetal growth because universal ultrasound screening for fetal growth restriction in the third trimester is not recommended [62].

In our study, we focused on FGR < 3rd percentile and have shown a significant association between elevated uterine artery Doppler PI and FGR. Altered uterine artery Doppler waveform in the first trimester has been described to be associated with FGR [49,52]. Peixoto et al. investigated the UA PI at mid-trimester with transvaginal ultrasound. They defined a reference range from 1.14 at 20 weeks to 0.95 at 24 weeks of gestation [63]. These findings comply with the results of another study by a prospective cohort study of 162 patients. They confirmed the prediction of hypertensive disorders and FGR with UA PI in the first trimester but could not show the UA PI as a predictive factor in the second trimester [64]. Velauthar et al. concluded in their meta-analysis involving 55,974 women that the specificity (93.1%, CI 90.6–95.0) of abnormal uterine artery Doppler for predicting early-onset fetal growth restriction is high, but the sensitivity (39.2%, CI 26.3–53.8) is low. The sensitivity of predicting FGR at any week of gestation was even lower (15.4%, CI 12.4–18.9), with almost the same specificity (93.3%, CI 90.9–95.1). Due to the inclusion of different articles in this meta-analysis, the definition of growth restriction could not be homogeneously defined [49]. We found a sensitivity of 52% with a specificity of 66% (AUC 0.6) for abnormal uterine artery Doppler PI for the prediction of FGR < 3rd percentile.

In our study, women with the combination of low PAPP-A and elevated uterine artery Doppler PI in the first trimester had a significantly higher risk of developing FGR < 3rd percentile than women with only one or no positive parameter. This is in accordance with the recently published findings of He et al. In contrast to our study, they described much better predictive values for FGR using the combination of PAPP-A and uterine artery Doppler in the first trimester. They described that the specificity was significantly improved by including serum PAPP-A levels in their calculations [25]. As they defined FGR as birth weight < 10th percentile, it cannot be excluded that their population consisted of a certain amount of small for gestational age fetuses.

Regarding the use of the nuchal translucency and ß-HCG serum levels for the prediction of FGR, we could not show any significant differences in our study. Our findings showed a reduced NT in pregnancies complicated by FGR compared to the control group. However, these differences did not reach statistical significance. In addition, we could not find a significant association between ß-HCG serum levels and the occurrence of FGR. The meta-analysis of Karagiannis et al. found decreased serum ß-HCG levels (0.89 vs. 0.97 MoM) and reduced NT (0.10 vs. 0.12 MoM) in pregnancies with SGA fetuses in comparison with Non-SGA pregnancies [46]. These findings have been prospectively confirmed by Poon et al. with a lower median NT (0.10 vs. 0.12 MoM) in SGA fetuses compared to the control group and also reduced ß-HCG serum levels (0.9 vs. 1.0 MoM) [45]. It should be emphasized that both studies examined patients diagnosed with SGA fetuses, which is defined as an EFW < 10th percentile. At the same time, our study investigated the prediction of FGR with EFW below the 3rd percentile, which complies with significantly higher rates of perinatal morbidity and mortality [65].

The definition of fetal growth restriction still presents an issue of international discussion. Diagnostic criteria are defined differently, depending on which guideline we refer [1,2,66,67]. Some guidelines include fetuses with EFW or abdominal circumference (AC) < 10th percentile [2,67]. The International Society of Ultrasound in Obstetrics and Gynecology classified the diagnosis in early and late-onset FGR. They define early-onset FGR as EFW or AC < 3rd percentile or under the 10th percentile in combination with pathologic PI of the uterine artery. Moreover, EFW or AC < 3rd percentile counts to the diagnostic criteria of late-FGR only in combination with other sonographic findings, including characteristics of fetometry and fetal blood flow velocities [66]. This definition results in a consensus on the Delphi procedure. The definition of fetal growth restriction by Gordjin et al. is different from the definition we used [1]. We used birth weight < 3rd percentile as the definition of FGR, which is still widely accepted [58,68].

International guidelines for the diagnosis and management of SGA fetuses and FGR mainly concentrate on the implementation of ultrasound. Therefore, no recommendations are proposed in the guidelines to use biomarkers such as PAPP-A for risk prediction [54,66,67,69]. Regarding the findings of the American College of Obstetricians and Gynecologists, the use of PAPP-A does not have a significant improvement in perinatal outcomes [67]. Lees et al. investigated the evidence of current recommendations of international guidelines on fetal growth restriction. According to this recent review, valid risk stratification assessment has not been introduced into clinical practice so far [68]. On the other hand, the Fetal Medicine Foundation designed a risk stratification tool to predict the development of FGR on the basis of a large prospective screening study including more than 30,000 pregnancies [45]. Karagiannis et al. established a risk calculation combining maternal history, PAPP-A, placenta-like growth factor (PlGF), uterine artery Doppler PI and mean arterial pressure. They conclude that with this risk calculation performed at 11–13 weeks, it was possible to detect 73% of SGA requiring delivery before 37 weeks and 46% of those delivering at term [46]. More effective prediction of FGR may be achieved by combined screenings in the second and third trimesters [70,71,72,73,74,75,76,77,78,79].

It is essential to evaluate the risk for pregnancy complications, such as FGR, and it might be unlikely that one single marker can predict precisely the risk of FGR. We identified low PAPP-A and high uterine artery Doppler PI in the first trimester to be associated with the development of FGR during pregnancy. Nevertheless, our results do not allow using PAPP-A and uterine artery Doppler PI as diagnostic or screening tests. Closer monitoring of fetal growth in pregnancies with altered PAPP-A and uterine artery Doppler PI, together with other markers, could improve pregnancy outcomes. This has to be evaluated in further studies.

Other approaches for screening tools in the first trimester include further examinations with 3D-ultrasound or magnetic resonance imaging (MRI) of the placental structure and/or volume to determine placental abnormalities and assess the association with FGR [19,80,81,82]. Ultrasound findings indicate umbilical artery PI Doppler as predictive value for FGR. Recent studies suggest compromising statements on risk assessment in patients with late-onset FGR by conducting umbilical vein blood flow [80]. However, the implementation of umbilical vein blood flow has not been investigated so far. Another approach to predicting fetuses small for gestational age was described by Gurgel Alves et al. The authors agreed with the general opinion that UA is valid as a predictor of SGA and preeclampsia in the first trimester. However, their findings did not show a significant association with maternal ophthalmic Doppler indices and SGA, either isolated or in combination with the UA, performed in the first trimester [83]. Further studies should be performed on new screening methods and on the combination of these parameters.

The identification of risk factors for developing FGR is essential for the establishment of prophylactic treatment. Although there has not been significantly effective management for the prevention of FGR, however, most international guidelines recommend 100mg of aspirin starting at gestational week 16 [54]. The prophylactic application of aspirin has been induced based on two meta-analyses observing a modest risk reduction of the occurrence of FGR [14,84]. These findings, however, are discussed as controversial and an issue of current research [85]. Rolnik et al. suggest the use of 150 mg aspirin as a single dose in the evening as a significant reduction of the occurrence of preeclampsia. However, they did not describe the effect on the outcome of FGR [86]. Additionally, Tan et al. demonstrated that in pregnancies identified as high-risk pregnancies for preterm preeclampsia by first trimester combined screening, the use of low-dose aspirin decreased the incidence of preterm FGR by about 40% and that of early FGR by 73%. The aspirin-related reduction in the incidence of SGA was mainly due to the decrease in pregnancies with PE. In patients without preeclampsia, the use of low-dose aspirin was not connected to a significant reduction in the incidence of FGR [87]. Perhaps future studies will be able to clarify these controversial findings.

This analysis has several strengths, including the high number of included pregnant women, the structured inclusion process and the defined ultrasound evaluations. Furthermore, we used the accepted definition of FGR as birth weight < 3rd percentile to exclude pregnancies with small for gestational age babies.

However, there were study limitations. We did not include measurements of the umbilical artery and middle cerebral artery Dopplers to identify fetuses with growth restriction. Other angiogenetic markers associated with FGR were not measured and could not be included in the final analysis.

## 5. Conclusions

In conclusion, PAPP-A levels and abnormal uterine artery Doppler PI in the first trimester are independent predictors of FGR < 3rd percentile, and they present a high negative predictive value.

## Figures and Tables

**Table 1:** Patient’s characteristics (n = 1930).

Characteristic	Results
Maternal age (years) ^a^	34.35 ± 4.56
Body mass index (kg/m^2^) ^a^	22.51 ± 3.40
Gestational age at birth (weeks) ^a^	39.15 ± 1.74
Birth weight (grams) ^a^	3304 ± 524
Fetal growth restriction below 3rd percentile ^b^	101 (5.2)
Primiparous ^b^	907 (47)

Numbers are given as ^a^ mean ± SD or ^b^ numbers (percent).

**Table 2:** Comparison between women with fetal growth-restricted fetuses (birth weight < 3% percentile) and normal birth weight fetuses.

Characteristic	FGR (n = 101)	Control Group (n = 1829)	*p*
Maternal age (years) ^a^	34.3 ± 4.4	34.2 ± 4.6	0.99
Body-mass-index (kg/m^2^) ^a^	22.8 ± 4.9	22.0 ± 3.2	0.33
Primiparous ^b^	29 (51.8)	166 (46.2)	0.47
Nuchal translucency (mm) ^a^	1.5 ± 0.3	1.7 ± 0.5	0.06
PAPP-A (MoM) ^a^	0.80 ± 0.38	1.15 ± 0.59	<0.001
ß hCG (MoM) ^a^	1.52 ± 0.94	1.36 ± 0.85	0.42
Uterine artery Doppler PI 1st trimester ^a^	1.82 ± 0.70	1.56 ± 0.47	0.01

FGR, fetal growth restriction (<3% percentile birth weight); PAPP-A, pregnancy-associated plasma protein A; ß hCG, beta-human chorionic gonadotropin; MoM, multiple of the median; mm, millimeter; PI, Pulsatility index. Numbers are given as ^a^ mean ± SD and the *t*-test for statistical significance or ^b^ numbers (percent) and the Chi-square test for statistical significance.

**Table 3:** Logistic regression analyses with the dependent variable fetal growth restriction (fetal birth weight percentile < 3%).

Characteristic	Regression Coefficient	SE	Wald	Exp(B)	95% CI	*p*
PAPP-A (MoM) at 11–14 wks	1.629	0.709	5.28	5.09	1.27–20.44	0.009
Uterine artery Doppler PI 1st trimester	−0.973	0.43	5.02	0.38	0.16–0.89	0.01

PAPP-A, pregnancy-associated plasma protein A; MoM, multiple of the median; wks, gestational weeks; PI, pulsatility index; SE, standard error; CI, confidence interval.

## Data Availability

The data presented in this study are available on request from the corresponding author. The data are not publicly available due to local data protection guidelines.
